# 1396. COVID-19 Hospitalization Impact of Age, Race and Ethnicity Among Medicaid and Uninsured

**DOI:** 10.1093/ofid/ofac492.1225

**Published:** 2022-12-15

**Authors:** Mamta K Jain, Yi Zhang, Mae Thamer, Michael Harms, Jillian Smartt, Stefanie Baierlipp, Kavita Bhavan

**Affiliations:** UT Southwestern Medical Center, Dallas, Texas; Medical Technology and Practice Patterns Institute, Bethesda, Maryland; Medical Technology and Practice Patterns Institute, Bethesda, Maryland; Parkland Health, Dallas, Texas; Parkland Health, Dallas, Texas; Dallas-Fort Worth Hospital Council Education and Research Foundation, Irwing, Texas; UT Southwestern Medical Center, Dallas, Texas

## Abstract

**Background:**

Texas has one of the highest rates of uninsured patients in the US and is one of the few states that has not expanded Medicaid (Fig 1). We sought to examine if there were differences by age, race, and ethnicity in the risk COVID-19 outcomes among those with Medicaid coverage vs. the uninsured population.

**Figure 1:**
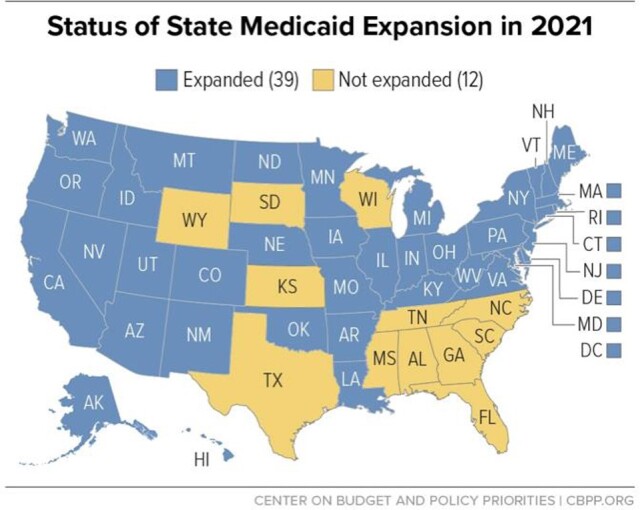
Medicaid Expansion States. Blue are the states which expanded Medicaid. Yellow are the states which did not. Texas is one of the largest non-Medicaid expansion states.

**Methods:**

We conducted a retrospective analysis of all patients hospitalized in 81 hospitals in Dallas-Fort Worth area. All inpatients with COVID-19 from 3/1/2020 to 9/30/2021 were included in the analysis. We examined the following COVID-19 outcomes: ICU care, pneumonia, and respiratory failure stratified (separate logistic models for each outcome) by race, ethnicity, and age adjusted for a multitude of sociodemographic, clinical, and co-morbid characteristics (Fig 2).

**Figure 2:**
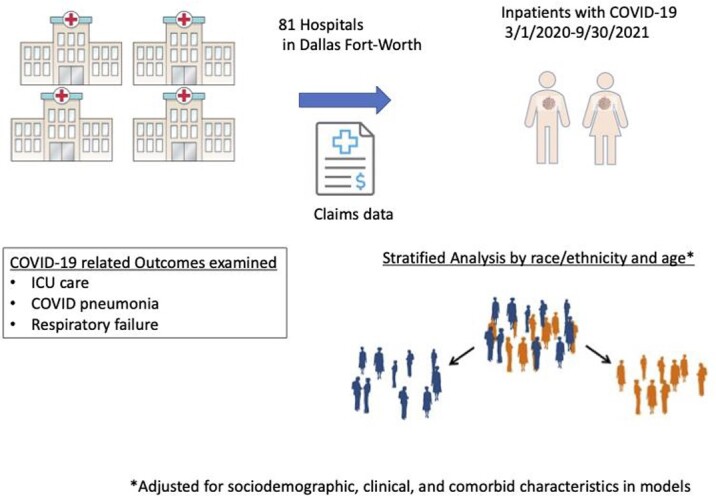
Graphic Methods

**Results:**

71,778 individuals diagnosed with COVID-19 were hospitalized: 12.9% had Medicaid and 23% were uninsured. For all COVID-19 study outcomes (ICU care, pneumonia, and respiratory failure), White Medicaid patients had lower odds ratios vs. their White uninsured counterparts indicating worse outcomes compared to Black Medicaid patients vs. Black uninsured counterparts (Table 1). Similarly, for all outcomes, Hispanic Medicaid patients had lower (worse) odds ratios vs. Hispanic uninsured counterparts compared to the same model with non-Hispanic patients. Finally, for all three outcomes, the youngest Medicaid age cohort (18-44 years) were less likely to require ICU care, have pneumonia or respiratory failure vs. the youngest uninsured patients; while conversely there was trend (not always statistically significant) that middle aged or older Medicaid cohorts were more likely compared to their same age uninsured counterparts to experience these outcomes.

**Table 1:**
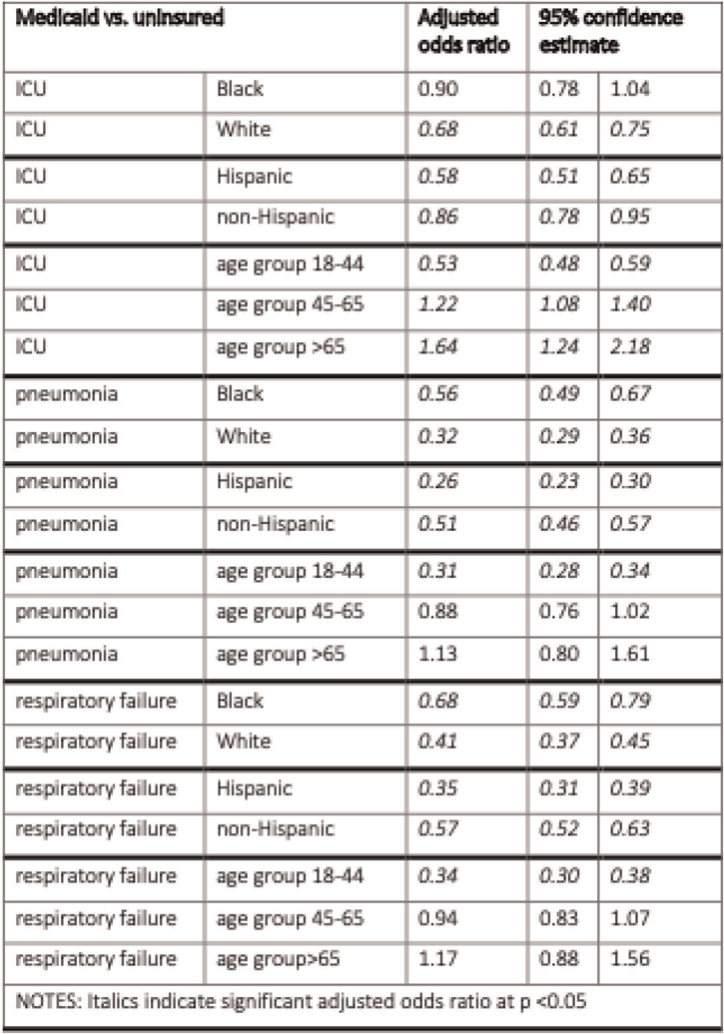

Adjusted odds ratio of COVID-19 outcomes by insurance status stratified by race, ethnicity, and age group.

**Conclusion:**

We found that age modified the risk for ICU care with younger Medicaid recipients at lower odds vs. uninsured than older cohorts. For race and Hispanic ethnicity, all Medicaid groups had lower likelihood of poor COVID-19 outcomes compared to their uninsured counterparts. However, the effect was more pronounced among Whites vs. Blacks and Hispanics vs. non-Hispanics (Fig 3). Providing health insurance such as Medicaid to uninsured younger patients could significantly improve health outcomes, especially among Whites, Hispanics, and younger patients.

**Figure 3:**
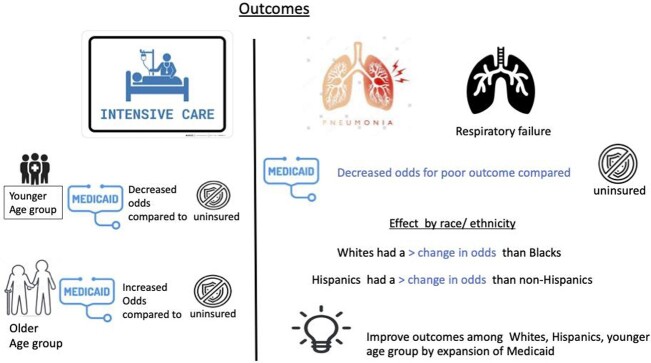
Graphic Conclusion

**Disclosures:**

**Mamta K. Jain, MD, MPH**, Gilead Sciences: Grant/Research Support|GSK/ViiV: Grant/Research Support|Janssen: Grant/Research Support|Merck: Grant/Research Support|Regeneron: Grant/Research Support **Mae Thamer, PhD**, Gilead: Grant/Research Support **Kavita Bhavan, MD, MPH**, Gilead: Grant/Research Support.

